# Identifying Robust Risk Factors for Knee Osteoarthritis Progression: An Evolutionary Machine Learning Approach

**DOI:** 10.3390/healthcare9030260

**Published:** 2021-03-01

**Authors:** Christos Kokkotis, Serafeim Moustakidis, Vasilios Baltzopoulos, Giannis Giakas, Dimitrios Tsaopoulos

**Affiliations:** 1 Institute for Bio-Economy & Agri-Technology, Center for Research and Technology Hellas, 60361 Volos, Greece; d.tsaopoulos@certh.gr; 2 Department of Physical Education & Sport Science, University of Thessaly, 38221 Trikala, Greece; ggiakas@gmail.com; 3 AIDEAS OÜ, Narva mnt 5, Tallinn, 10117 Harju maakond, Estonia; s.moustakidis@aideas.eu; 4 Research Institute for Sport and Exercises Sciences, Liverpool John Moores University, Liverpool L3 3AF, UK; V.Baltzopoulos@ljmu.ac.uk

**Keywords:** knee osteoarthritis prediction, feature selection, genetic algorithm, machine learning, explainability

## Abstract

Knee osteoarthritis (KOA) is a multifactorial disease which is responsible for more than 80% of the osteoarthritis disease’s total burden. KOA is heterogeneous in terms of rates of progression with several different phenotypes and a large number of risk factors, which often interact with each other. A number of modifiable and non-modifiable systemic and mechanical parameters along with comorbidities as well as pain-related factors contribute to the development of KOA. Although models exist to predict the onset of the disease or discriminate between asymptotic and OA patients, there are just a few studies in the recent literature that focused on the identification of risk factors associated with KOA progression. This paper contributes to the identification of risk factors for KOA progression via a robust feature selection (FS) methodology that overcomes two crucial challenges: (i) the observed high dimensionality and heterogeneity of the available data that are obtained from the Osteoarthritis Initiative (OAI) database and (ii) a severe class imbalance problem posed by the fact that the KOA progressors class is significantly smaller than the non-progressors’ class. The proposed feature selection methodology relies on a combination of evolutionary algorithms and machine learning (ML) models, leading to the selection of a relatively small feature subset of 35 risk factors that generalizes well on the whole dataset (mean accuracy of 71.25%). We investigated the effectiveness of the proposed approach in a comparative analysis with well-known FS techniques with respect to metrics related to both prediction accuracy and generalization capability. The impact of the selected risk factors on the prediction output was further investigated using SHapley Additive exPlanations (SHAP). The proposed FS methodology may contribute to the development of new, efficient risk stratification strategies and identification of risk phenotypes of each KOA patient to enable appropriate interventions.

## 1. Introduction

Knee osteoarthritis (KOA) has a higher prevalence rate compared with other types of osteoarthritis (OA). KOA is a consequence of mechanical and biological factors. Specifically, this complex interplay includes joint integrity, genetic predisposition, biochemical processes, mechanical forces and local inflammation. At the onset of this disease, the main consequences are low quality of life due to pain, social isolation and poor psychological state. According to the literature, age, obesity and previous injuries due to sports or occupational/daily activities show a high correlation with KOA [1,2,3,4]. Particular reference should be made to the specificity of this disease. Specifically, the knee osteoarthritic process is gradual, with a variation in symptom frequency, patterns and intensity [5,6]. Despite the constant effort of the scientific community, research on KOA prediction is still necessary to investigate and explore the multifactorial nature of the disease.

One of the main challenges is the development and refinement of prognostic KOA models that will be applicable to the entire population. In this effort, an increase has been observed in the number of studies using artificial intelligence techniques due to the existence of big data [7,8,9,10,11,12]. As a result of this, several techniques have been reported in the literature in which feature selection (FS) techniques and machine learning (ML) models were used to predict KOA [13,14]. There are several studies where heterogenous datasets were considered including symptoms and nutrition questionnaires, medical imaging outcomes, subject characteristics and behavioral and physical exams. Lazzarini et al. used a guided iterative feature-elimination algorithm and principal component analysis (PCA) and they demonstrated that it is possible to accurately predict the incidence of KOA in overweight and obese women using a small subset of the available information [15]. Specifically, they achieved their aim by using only five variables and Random Forest (RF) with an area under the curve (AUC) of 0.823. In another study, Du et al. used PCA and four well-known ML models to predict the change of Kellgren and Lawrence, joint space narrowing on the medial compartment and joint space narrowing on the lateral compartment grades by using magnetic resonance imaging (MRI) [16]. They demonstrated that there are more informative locations on the medial compartment than on the lateral compartment. They achieved an AUC of 0.695–0.785. Furthermore, Halilaj et al. built a model to predict long-term KOA progression taking into account self-reported knee pain, radiographic assessments of joint space narrowing from the Osteoarthritis Initiative (OAI) database and least absolute shrinkage and selection operator (LASSO) regression models [17]. In this task, an AUC of 0.86 for radiographic progression was achieved on a 10-fold cross-validation scheme. In another study, Pedoia et al. used topological data analysis as a feature engineering technique in combination with MRI and biomechanics multidimensional data [18]. In the attempt to meet the existing gap in multidimensional data analysis for early prediction of cartilage lesion progression in KOA, they used logistic regression as the ML model, achieving an AUC of 0.838. Moreover, in the task of predicting KOA severity, Abedin et al. made use of elastic net regression and were able to (i) identify the variables that have high predictive power and (ii) quantify the contribution of each variable with an overall root mean square error (RMSE) of 0.97 [19].

In 2019, Nelson [20] et al. applied an innovative ML approach in order to identify key variables associated with a progression phenotype of KOA. Specifically, they combined distance-weighted discrimination algorithm, direction-projection-permutation testing and clustering methods to identify phenotypes that are potentially more responsive to interventions. Another study by Widera et al. was based on recursive feature elimination that selects the best risk factors for the prediction of KOA progression from incomplete imbalanced longitudinal data [21]. They used five ML models achieving F1 scores from 0.573 up to 0.689. Furthermore, Tiulpin et al. applied a multi-modal ML-based KOA progression prediction model which utilizes baseline characteristics, clinical data, radiographic assessments and the probabilities of KOA progression that are calculated from a deep convolutional neural network [22]. To handle the heterogeneity of the available data, they applied a gradient boosting machine classifier with an AUC of 0.79–0.82. Moreover, Kokkotis et al. presented a robust FS approach that could identify important risk factors in a KOA prediction task [10]. The novelty of this approach lies in the combination of well-known filter, wrapper and embedded techniques, whereas feature ranking is decided on the basis of a majority vote scheme to avoid bias. A 74.07% classification accuracy was achieved by support vector machines. In addition, Jamshidi et al. worked on the identification of important structural KOA progressors [23]. They used six FS models and the best classification accuracy was achieved by multi-layer perceptron (MLP, AUC = 0.88 and 0.95 for medial joint space narrowing at 48 months and Kellgren–Lawrence (KL) grade at 48 months, respectively). In another study, Wang et al. employed a long short-term memory model to predict KOA progression [24]. They used observed time series (5-year clinical data from OAI) and they predicted the KL grade with 90% accuracy. Despite all the aforementioned valuable contributions, few of the above studies have attempted to apply robust FS methodologies for the development of ML models for the prediction of KOA progression [13]. Therefore, there is still a significant knowledge gap on the contribution of clinical data on KOA progression prediction and their impact on the training of the associated ML predictive models.

Due to the multidimensional and imbalanced nature of the datasets that are publicly available for KOA, robust identification of the best features for the prediction of KOA is a challenging task. According to our knowledge, only few studies [25,26,27] have attempted to address the complicated interaction of the aforementioned challenges (high dimensionality and data imbalance) in biomedical datasets (but none in the area of KOA). Main examples of FS methods that were applied in various fields to overcome the imbalance problem are (a) resampling techniques [28,29,30,31], (b) ensemble learning techniques [25,32,33], (c) cost-sensitive learning [34,35], (d) one-class learning [36,37] and (e) active learning [38,39]. Hence, to cope with the aforementioned FS challenges (high dimensionality and data imbalance), we propose an FS technique that incorporates a number of characteristics towards the identification of robust risk factors that generalize well over the whole dataset. The proposed FS methodology, termed GenWrapper in this paper, is an evolutionary genetic algorithm (GA)-based wrapper technique that differentiates from the classical GA-based FS techniques in terms of the following: (i) GenWrapper applies random under-sampling at each individual solution, forcing the GA to converge to solutions (feature subsets) that generalize well regardless of the applied data sampling; (ii) It ranks features with respect to the number of times that they have been selected in all the individual solutions for the final population. The combined effect of the aforementioned GenWrapper characteristics leads to selected features that consistently work well at any possible data sample and, thus, have increased generalization capacity with respect to KOA progression. An extensive comparative analysis has been performed to prove the superiority of GenWrapper over well-known FS algorithms with respect to both prediction accuracy and generalization.

The rest of the paper is organized as follows. Section 2 presents the proposed methodology including a short description of the dataset and the associated KOA prediction problem. The employed data pre-processing, FS approach, validation strategy applied along with an AI explainability analysis are also given in Section 2. Section 3 includes the results of the suggested FS, presents the selected risk factors, compares the performance of GenWrapper with well-known FS algorithms and, finally, provides an analysis with respect to the impact of the most important features to the prediction outcome. Discussion of the main results is provided in Section 4 and conclusions are drawn in Section 5.

## 2. Methods

### 2.1. Dataset Description

Data were obtained from the Osteoarthritis Initiative (OAI) database (available upon request at https://nda.nih.gov/oai/, accessed on 18 June 2020), which include clinical evaluation data, a biospecimen repository and radiological (magnetic resonance and X-ray) images from 4796 women and men aged 45–79 years. The features considered in this work for the prediction of KL are shown in Table 1. The current study included clinical data from the baseline and the first follow-up visit at month 12 from all individuals being at high risk to develop KOA or without KOA. Specifically, the dataset contains 957 features from eight different feature categories, as shown in Table 1. In addition, our study was based on the Kellgren and Lawrence (KL) grade as the main indicator for assessing the OA clinical status of the participants. Specifically, the variables “V99ERXIOA” and “V99ELXIOA” were used to assign participants into subgroups (classes) of participants whose KOA status progressed or not.

### 2.2. Problem Definition

**Figure 1:**
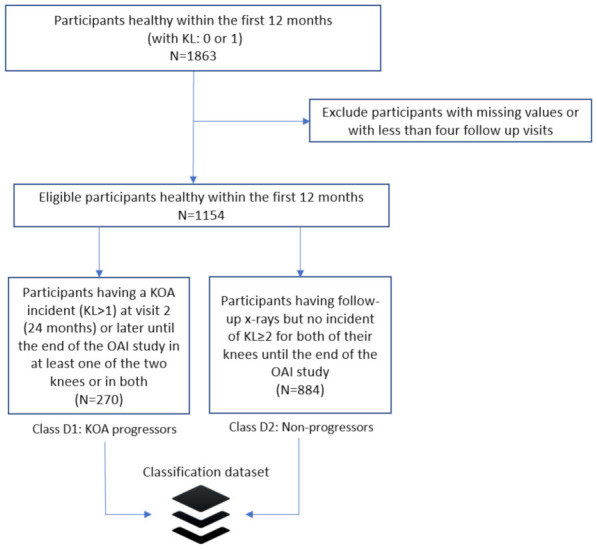
Stratification of the patients in our study and formulation of the training dataset. Inclusion/exclusion criteria are presented along with the definition of the two data classes (knee osteoarthritis (KOA) progressors and non-progressors).

In this paper, we consider KL grade prediction as a two-class classification problem. Specifically, the participants of the study were divided into two groups: (a) Non-progressors—healthy participants with KL0 or 1 at baseline with no further incidents in both of their knees until the end of the OAI data collection; (b) KOA progressors—participants who were healthy during the first 12 months (with no incident at baseline and the first visit) and then they had an incident (KL ≥ 2) recorded at their second visit (24 months) or later, until the end of the OAI study (Figure 1). 

### 2.3. Data Pre-Processing

Initially, data cleaning was performed by excluding the columns with more than 20% missing values compared to the total number of subjects. Afterwards, data imputation was performed to handle missing values. As an imputation strategy, mode imputation was implemented to replace missing values of the numerical or categorical variables by the most frequent value of the non-missing variables [40]. Standardization of a dataset is a common requirement for many ML estimators [41]. In our paper, data were normalized by removing the mean and scaling to unit variance to build a common basis for the machine learning algorithms that followed. After application of the exclusion criteria, classes 1 (KOA progressors) and 2 (non-progressors) comprised 270 and 884 samples, respectively.

### 2.4. Feature Selection

Class imbalance is among the major challenges encountered in health-related predictive models, skewing the performance of ML algorithms and biasing predictions in favor of the majority class. To alleviate this problem, a novel evolutionary feature selection is proposed in this paper that overcomes the class imbalance problem and increases the generalization capacity of the finally employed ML algorithm.

**Figure 2:**
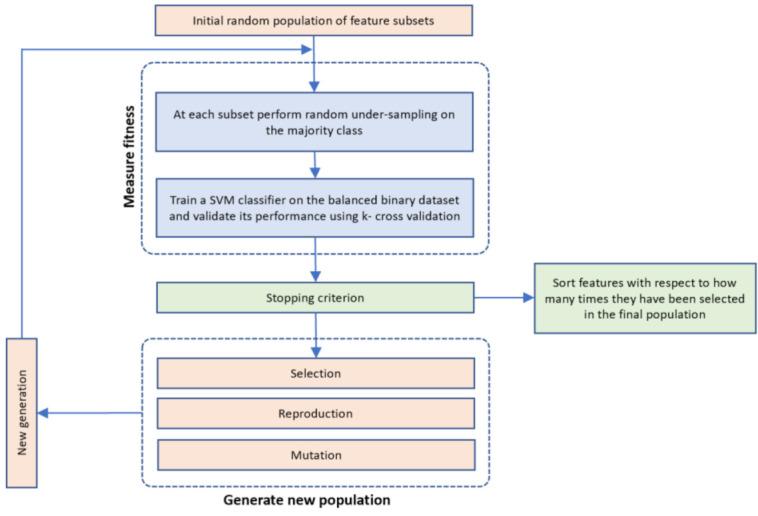
The proposed GenWrapper feature selection (FS) methodology that includes all the involved processing steps: (i) generation of the initial population; (ii) fitness measurement approach; (iii) stopping criterion; (iv) evolution mechanisms and (v) final feature ranking after the termination of the genetic algorithm (GA).

The proposed FS is a genetic algorithm-based approach inspired by the procedures of natural evolution (Figure 2). It operates on a population of individuals (solutions), and at each generation, a new population is created by selecting individuals according to their level of fitness in the problem domain (KOA progression in our case). The individuals are then recombined using operators borrowed from natural genetics (selection, reproduction and mutation). This iterative process leads to the evolution of populations of individuals that are better suited to the problem domain. Here, each individual in the population represents an ML model trained on a specific feature subset to discriminate the aforementioned classes (KOA progressors versus non-progressors). Genes are binary values and represent the inclusion or not of particular features in the model. The number of genes is the total number of input variables in the dataset. Concatenating all genes, a so-called individual or chromosome is formulated that represents a possible solution (feature subset) in our FS problem.

**Figure 3:**
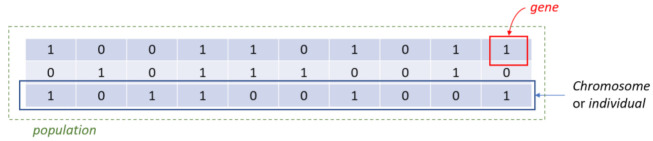
Definition of genes, chromosomes and population.

The Optimization Toolbox of MATLAB 2020b was used for the implementation of GenWrapper. The proposed FS algorithm proceeds along the following steps:

- Step 1. Initialization
  A group of k chromosomes are randomly generated, forming the initial population of individuals.
- Step 2. Fitness assignment
  A fitness value is assigned to each chromosome in the population. Specifically, the process of measuring fitness in GenWrapper can be summarized as follows. The following 3-step process (Figure 3) is repeated for each of the chromosomes of the population:
  Step 2.1. From the training dataset, we keep only the features that have a value of 1 in the current chromosome. This creates a truncated training set.
  Step 2.2. Random undersampling on the majority class is performed on the truncated training set. This action leads to a balanced variant of the truncated training set.
  Step 2.3. A classifier is trained on the newly produced balanced dataset. Linear support vector machines (SVMs) have been chosen as the main classification criterion due to their generalization capability.
  Step 2.4. A k-fold cross-validation scheme is employed to validate the classifier performance that is finally assigned as a fitness value to the specific individual.
- Step 3. Termination condition
  The algorithm stops if the average relative change in the best fitness function value over Κ generations is less than or equal to a pre-determined threshold.
- Step 4. Generation of a new population
  In case the termination criterion is not satisfied, a new population of individuals is generated by applying the following three GA operators:
  Selection operator: The best individuals are selected according to their fitness value.
  Crossover operator: This operator recombines the selected individuals to generate a new population.
  Mutation operator: Mutated versions of the new individuals are created by randomly changing genes in the chromosomes (e.g., by flipping a 0 to 1 and vice versa).
- Step 5. The algorithm returns to step 2.
- Step 6: Final feature ranking determination
  Upon termination of the GA algorithm, the features are ranked with respect to the number of times that they have been selected in all the individuals (chromosomes) of the final population.
  Step 6.1. A feature gets a vote when it has a value of 1 in a chromosome of the final generation.
  Step 6.2. Step 6.1 is repeated for all the chromosomes of the final generation and the features’ votes are summed up.
  Step 6.3. Features are ranked in descending order with respect to the total number of votes received.

**Figure 4:**
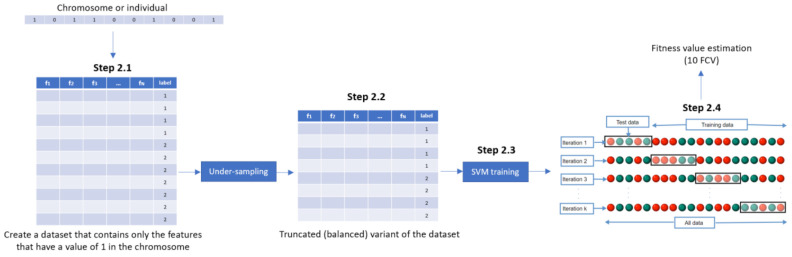
Proposed mechanism for estimating the fitness of each chromosome within a generation.

GenWrapper evaluates the fitness of each chromosome (feature subset) by firstly applying random undersampling at the associated dataset (in step 2.2) and then by training an SVM classifier on it (Figure 4). The k-fold cross-validation (CV) performance of the SVM is considered as the fitness of the specific individual. The best individuals (feature subsets that maximize the fitness value) are then selected and combined to generate the new population. This procedure forces the GA to converge to solutions (feature subsets) that generalize well regardless of the specific sampling that has been applied. If a specific resampling process had been applied universally on the dataset before the application of the GA-based FS, then this would lead to overfitting, since the GA algorithm would try to select the best features that fit to the specific data sample. The proposed technique integrates a random sampling mechanism when evaluating each individual, leading to features that generalize well on the whole population. Moreover, the choice of k-fold cross-validation as a validation scheme guarantees that the selected features have high predictive capacity over the whole dataset considered. Another characteristic of the proposed evolutionary FS is the way that features are selected/ranked in the final population. Instead of selecting features from the best individual in the final population, the proposed selection criterion relies on the general performance of features over the whole final population. The best solution (the one with the highest fitness value in the final population) corresponds only to the maximum possible accuracy that can be achieved by a selected feature subset on a specific subset of the whole sample. However, this does not necessarily mean that the best solution generalizes well in the whole sample. Therefore, to achieve the best possible generalization, the proposed FS ranks features with respect to the number of times that they have been selected in all the individuals of the final population. The parameters of the proposed GA-based FS have been properly selected and are cited in Table 2 below.

### 2.5. Learning

Given that the main objective of paper is the identification of robust risk factors, two well-known linear ML models (linear regression (LR) and linear SVM) were utilized to evaluate the predictive capability of the selected features. The reason for employing linear models is because (i) they are computationally efficient, so they can be executed multiple times within a repetitive process such as the GA-based algorithm that is proposed in this paper, and (ii) they generalize well and, therefore, can be used to assess the generalization performance of the selected features. A brief description of these models is given below.

LR is the most commonly used algorithm for solving classification problems [42]. It is an extension of the linear regression model for classification problems and it models the probabilities for classification problems with two possible outcomes. SVMs are supervised learning models for classification, regression and outlier detection but are more commonly used in classification problems [43]. SVMs are effective in high-dimensional spaces and are still effective in cases where the number of dimensions is greater than the number of samples.

### 2.6. Validation

To evaluate the predictive capacity of the selected feature subset, a repeated cross validation process was adopted using the aforementioned classifiers. Specifically, the validation approach proceeds with the following steps

- Step 1. Random undersampling is applied on the majority class, and the retained samples along with those from the minority class form a balanced binary dataset.
- Step 2. A classifier is built on the balanced binary dataset and its accuracy is calculated using 10-fold cross-validation (10FCV).
- Step 3. Steps 1 and 2 are repeated 10 times, each one using a different randomly generated balanced dataset.
- Step 4. The final performance is calculated by averaging the obtained 10FCV classification accuracies. The resulting final performance will be referred to here as mean 10FCV.

By adopting this repeated validation approach, we guarantee that the selected features are not only suitable for a specific data sample but that they generalize well over the whole dataset. The calculated mean 10FCV performance aggregates the accuracies from 100 training runs (10 repetitions of 10FCV) on different randomly created data samples, forming a reliable measure for estimating the predictive capacity of the selected features.

### 2.7. Explainability

To further assess the impact of the selected features on the classification outcome, SHapley Additive exPlanations (SHAP) were considered. SHAP is a game theoretic approach that explains the output of any machine learning model and achieves the connection of the optimal credit allocation with local explanations using the classic Shapley values from game theory that come with desirable properties [44]. In this study, Kernel SHAP is used, which is a specially weighted local linear regression to estimate SHAP values for any model (e.g., SVM in a two-class classification problem). The optimization of loss function L in Kernel SHAP is described below (in Equation (1)), where g is the explanation linear model that is trained on training data Z, $f\left(:\right)$ is the original prediction function to be explained and $z^{\prime }$ is a vector of 1s and 0s called coalition. Here, 1s indicate the presence of the corresponding feature, while 0 indicates its absence. $h_{x}\left(z^{\prime }\right)$ maps a feature coalition to a feature set on which the model can be evaluated, whereas $\pi_{x}\left(z^{\prime }\right)$ is the SHAP kernel.

(1) $$\left(f,g,\pi_{x}\right)\;=\underset{z^{\prime }\in Ζ}{\sum }{\left[f\left(h_{x}\left(z^{\prime }\right)\right)-g\left(z^{\prime }\right)\right]}^{2}\pi_{x}\left(z^{\prime }\right)$$

## 3. Results

In this section, we demonstrate the efficiency of the proposed feature selection algorithm in comparison with other well-known FS techniques. The most significant risk factors, as selected by the proposed FS methodology, are also presented, whereas their impact on the classification result is discussed employing SHAP.

### 3.1. Selection Criterion

**Figure 5:**
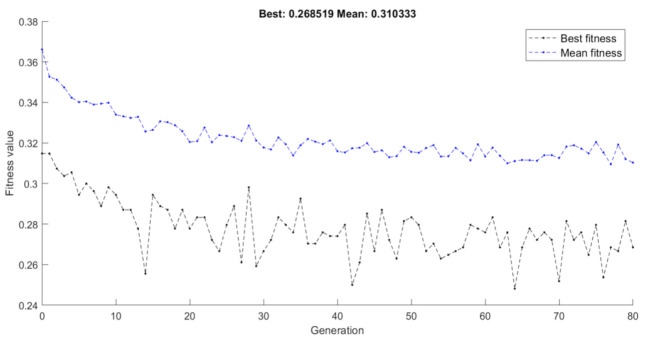
Fitness with respect to number of generations for GenWrapper. The black and blue dashed lines show the best and the mean fitness achieved at each generation, respectively.

Figure 5 shows the evolution of the proposed fitness value with respect to the number of generations. As it was discussed in Section 2, the mean fitness value is calculated by averaging the fitness values of all the 50 individual solutions in each generation. Each individual fitness value represents the performance of the employed ML model (SVM in our case) on a new, randomly generated balanced dataset (after downsampling the majority class) using k-fold cross-validation. Thus, the mean fitness value aggregates the performance of 50 employed ML models that were trained on slightly different versions of the initially available dataset. As it is observed in Figure 5, the mean fitness value decreases with the number of generations, meaning that the FS converges to a pool of selected feature subsets that have increased classification capacity, regardless of any specific data sampling.

**Figure 6:**
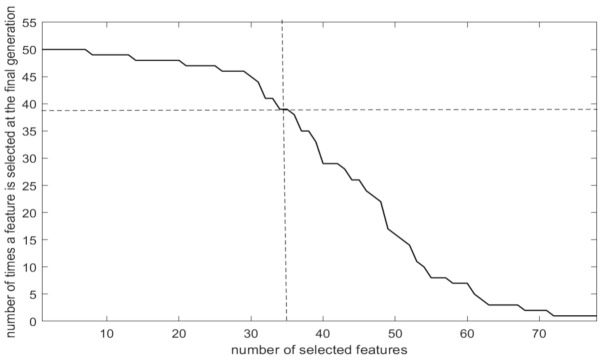
Feature ranking produced by the proposed FS (the dashed line indicates the number of features that were finally selected).

The dashed black line in Figure 5 represents the minimum fitness values received at each generation of the algorithm. However, as it was noted in Section 2, the best fitness value (0.26818 in our case) corresponds to a selected feature subset that has been decided based on its performance on a part of the available sample. The proposed scheme, instead of selecting the “best” feature subset of the final generation, proceeds by ranking the available features with respect to the times they have been selected in the 50 different individual solutions of the final generation. Figure 6 illustrates an example of such a ranking where seven features have been selected in all 50 individual solutions, another nine have been selected in 49 individual solutions and so on. The highly ranked features are the ones that are consistently selected by all individual solutions that are generated on different data samples.

To prove the superiority of the proposed feature selection criterion over the “best” individual solution, we performed the following experimentation. Two competing feature subsets were initially extracted: (a) the proposed one that has been selected after selecting the top 35 highly ranked features and (b) the feature subset extracted from the “best” individual solution of the final GA generation (comprising 42 features). The generalization capacity of both features subsets was assessed by employing the repetitive validation approach proposed in this paper and the results are shown in Table 3. The proposed feature ranking led to higher accuracy (in terms of mean performance, minimum and maximum accuracies), employing less features (35) compared to the ones selected in the “best” individual solution (42).

### 3.2. Features Selected

Table 4 cites the 35 features selected by the chosen GenWrapper FS approach. A short description of the features and the categories in which they belong are presented. Seven out of the 35 selected risk factors come from the symptoms category, representing parameters related to pain, swelling, stiffness and knee difficulty, demonstrating the relevance of symptoms in the occurrence and progression of KOA. Moreover, eight features represent diet and nutrition-related parameters that also constitute an important risk factor category. Nine of the features are related to physical activity or exams, whereas another five behavioral risk factors were selected as relevant to KOA progression. Medical history or status estimated through subjective (three self-reported risk factors) or more objective metrics (medical imaging outcomes such as the existence of osteophytes) were also selected by the proposed FS approach. Finally, two parameters describing subject characteristics were among the selected risk factors (specifically the patient’s body mass index (BMI) and height).

### 3.3. Comparative Analysis

The performance of the proposed FS methodology was compared with eight well-known FS techniques in the recent literature. The selected techniques along with their main characteristics are briefly presented below.

A classical wrapper FS was employed in which the feature selection process is based on a specific machine learning algorithm that we are trying to fit on a given dataset. It follows a time-consuming search approach by evaluating all the possible combinations of features against the evaluation criterion. The evaluation criterion is simply a performance measure which depends on the type of problem. Infinite latent feature selection (ILFS) is a probabilistic latent feature selection approach that performs the ranking step by considering all the possible subsets of features, bypassing the combinatorial problem [45]. Unsupervised graph-based filter (Inf-FS) is another FS algorithm proposed, again, by Roffo et al. (2015) [46]. In Inf-FS, each feature is a node in a graph, a path is a selection of features and the higher the centrality score, the most important the feature. It assigns a score of importance to each feature by taking into account all the possible feature subsets as paths on a graph. Correlation-based feature selection (CFS) sorts features according to pairwise correlations [47], whereas LASSO, proposed by Hagos et al. (2017), applies a regularization process that penalizes the coefficients of the regression variables while setting the less relevant ones to zero with respect to the constraint on the sum [48]. In LASSO, FS is a consequence of this process, when all the variables that still have non-zero coefficients are selected to be part of the model. Minimum redundancy maximum relevance (Mrmr) [49] is another well-known FS algorithm that systematically performs variable selection, achieving a reasonable trade-off between relevance and redundancy. A hybrid FS methodology was also employed that combines the outcomes of six FS techniques: two filter algorithms (Chi-square and Pearson correlation), three embedded ones (LightGBM, logistic regression and random forest) and one wrapper (with logistic regression) [10]. In this approach, all six FS techniques are applied separately, with each one resulting in a selected FS, and the final feature ranking is decided on the basis of a majority vote scheme. PCA is a well-known feature reduction method that reduces the dimensionality of data by geometrically projecting them onto lower dimensions called principal components (PCs), with the goal of finding the best summary of the data using a limited number of PCs. The MATLAB-based feature selection library FSLib 2018 (https://www.mathworks.com/matlabcentral/fileexchange/56937-feature-selection-library, accessed on 30 January 2021) was used for the implementation of the competing FS algorithms on a research workstation with Intel Core i7-7500 processor, 2.70 GHz CPU (16 GB RAM).

**Figure 7:**
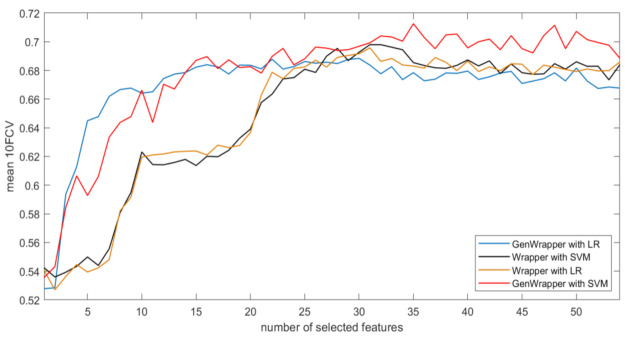
Accuracy (mean 10-fold cross-validation (10FCV)) with respect to selected features (curves): GenWrapper versus a classical wrapper using two classifiers (support vector machine (SVM) and logistic regression (LR)).

Figure 7 depicts the results of the comparison between the proposed GenWrapper FS and a classical wrapper FS technique. Specifically, the obtained mean 10FCV accuracies are shown with respect to the number of features as they have been ranked by the two compared approaches using two classifiers (LR and SVM). The following remarks can be extracted from Figure 7:

- GenWrapper significantly outperforms the classical wrapper FS, especially for a small number of selected features (up to 20). This superiority is proven for both SVM and LR;
- GenWrapper employing SVM gives the best overall performance (71.25% at 35 selected features).

**Figure 8:**
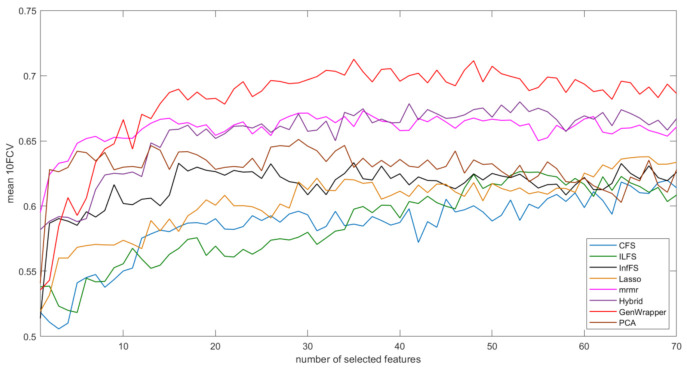
Accuracy (mean 10FCV) with respect to selected features: GenWrapper versus the remaining competing FS techniques. SVM was used for the classification task for all eight FS techniques.

Figure 8 shows the progression of the mean 10FCV accuracy with respect to the number of selected features for the proposed FS and the other seven competing FS techniques (CFS, ILFS, Inf-FS, Lasso, Mrmr, PCA and hybrid). In this comparative analysis, a linear SVM classifier were employed by all techniques since it proved to be the most efficient ML model. GenWrapper is the best-performing technique, achieving high accuracies (3.4% higher than the second best). Hybrid FS and Mrmr were the second and third best performers, achieving accuracies of 67.85% and 67.29%, respectively. Mrmr was very successful at the first 10 selected features but then it reached a threshold within the range of 67–68%, whereas the inclusion of further features had a minor or even negative effect on the classification performance. The rest of the FS techniques had moderate performances (61.97–65.11%). Table 5 also shows the best accuracies achieved by each technique and the number of features for which the best accuracy was achieved. GenWrapper achieved its best accuracy at a relatively small number of features (35), whereas the rest had inferior performances and, in most of the cases, at a higher number of features. The classical wrapper FS was the only one that selected slightly less features (31). A statistical comparison was finally conducted, verifying that the accuracies obtained by the proposed GenWrapper were significantly different (higher) to the ones of all the competing FS algorithms (*p* < 0.001).

**Figure 9:**
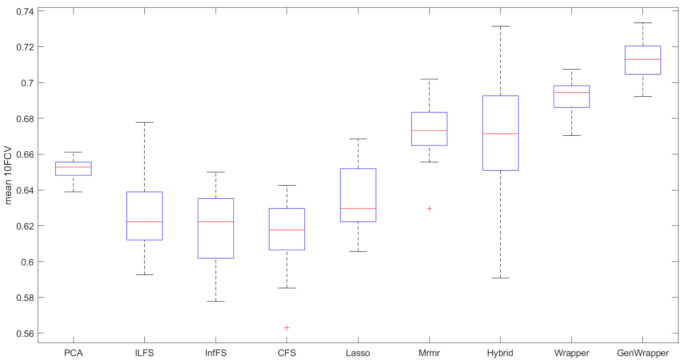
Bar graph comparison for the best models (SVMs trained on the optimum number of selected features per case). Red lines correspond to the mean 10FCV, blue boxes visualize the standard deviation of the obtained accuracies, dashed black lines show the min–max range and the red crosses depict outliers (if any).

The last part of the conducted comparative analysis focuses on a different performance metric—that is, the consistency of the obtained accuracies during the proposed repetitive validation process. As explained in the previous sections, the predictive capacity of the selected features is validated multiple times (10). In each of the ten repetitions, 10FCV is employed on a different, randomly selected balanced data sample. A feature subset could be considered as robust when it consistently leads to high accuracies over the ten repetitions. Figure 9 is a bar graph that visualizes (i) the mean 10FCV accuracies, (ii) the standard deviation of the 10FCV accuracies, (iii) the range ([min,max]) of the 10FCV accuracies and (iv) any outliers that deviate from the distribution of the 10FCV accuracies. GenWrapper was the most accurate approach (71.25%) and, at the same time, it proved to be the most consistent FS technique, with the great majority of obtained 10FCV accuracies being higher than 70%. The classical wrapper FS was also consistent over the ten repetitions but it was considerably less effective than the proposed GenWrapper. It should be noted that the hybrid FS approach achieved accuracies up to 72.5%; however, it does not generalize well given that it leads to a quite enlarged min–max range as well as an increased standard deviation, with the minimum accuracy being less than 60%. Mrmr has led to both moderate mean accuracy and moderate consistency (ranging between 66% and 70%) over the repetitions of the employed validation process. The rest of the competing FS approaches led to much lower 10FCV accuracies that ranged between 58% and 68%.

### 3.4. Explainability Results

**Figure 10:**
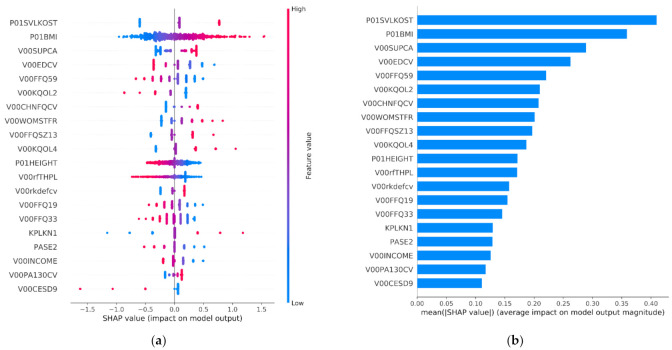
This figure depicts: (**a**) the SHAP summary plot and; (**b**) the SHAP feature importance for the SVM trained on the features selected by the proposed GenWrapper.

Figure 10a illustrates the features’ impact on the output of the final model (SVM) on the OAI dataset. It sorts features by the sum of SHAP value magnitudes over all samples and uses SHAP values to show the contribution of each feature (positive or negative) on the model’s output. The color represents the feature value (blue—low; red—high). This reveals, for example, that a high P01BMI (body mass index of the participants) increases the predicted status of the participants. Similarly to BMI, the features P01SVLKOST, V00SUPCA, V00CHNFQCV, V00WOMSTFR, V00FFQSZ13, V00KQOL4, V00rkdefcv, KPLKN1 and V00PA130CV have a positive effect on the prediction outcome (their increase drives the output to increase), whereas the rest have the opposite effect. Figure 10b demonstrates the mean absolute value of the SHAP values which represents the SHAP global feature importance. It should be noted that the features P01SVLKOST, BMI, V00SUPCA and V00EDCV were the most important variables that significantly affected the prediction output (Appendix A).

## 4. Discussion

Predicting KOA onset and its further progression is among the best strategies to reduce the burden of the disease. Risk factors for incident OA may differ from those for OA progression given that the incidence and progression of radiographic knee OA may involve different processes [50,51]. Several risk factors have been reported to be associated with the incidence of knee OA [1,52,53]. However, our understanding about predictive risk factors associated with KOA progression is limited due to the fact that the number of studies, in which risk factors and incidence of knee OA have been investigated longitudinally, is relatively small. This study contributes to the identification of robust risk factors for knee OA progression as a first, but very important, step toward achieving the goal of developing preventive strategies and intervention programs and finally reducing the incidence and associated morbidity of knee OA.

Identifying important features from an imbalanced data set is an inherently challenging task, especially in the current KOA prediction problem with limited samples and a massive number of features. Feature selection algorithms employing data resampling have been typically utilized to reduce the feature dimensionality and at the same time to overcome the class imbalance challenge. Oversampling algorithms randomly replicate examples from the minority class which in some scenarios can facilitate the FS process but is also prone to overfitting [54]. In data under-sampling, examples from the majority class are randomly discarded in order to rectify the disparities between classes. However, informative samples might be discarded from the final training set, reducing the generalization capabilities of the finally selected risk factors. New approaches are needed to address the intersection of the high dimensionality and imbalanced class problems due to their complicated interactions.

To cope with all the aforementioned challenges, the proposed FS technique incorporates a number of features aiming towards the identification of robust risk factors (with increased generalization capacity) extracted from a highly imbalanced dataset. GenWrapper relies on a stochastic method for function optimization based on the mechanics of natural genetics and biological evolution. This stochastic search is employed to identify a globally optimal feature subset, compared to a costly search that makes local decisions. The proposed FS performs better than traditional feature selection techniques, can manage datasets with many features and does not need any specific knowledge about the problem under study. Compared to traditional GA-based FS algorithms, GenWrapper applies random undersampling at each individual solution, forcing the GA to converge to solutions (feature subsets) that generalize well regardless of the applied data sampling. K-fold cross-validation is utilized to measure the fitness of each individual solution, guaranteeing that the selected features have high predictive capacity over the whole dataset considered. Finally, instead of selecting the “best” individual of the final population, the proposed FS ranks features with respect to the number of times that they have been selected in all the individual solutions of the final population. This leads to selected features that consistently work well at any possible data sample and, thus, have increased generalization capacity with respect to KOA progression.

Linear classifiers were employed on this study, and this choice can be attributed to the fact that evidence of linear separability between the two classes (progressors versus non-progressors) was identified in previous studies of the authors on the same problem. Specifically, as it was reported in [10], LR and linear SVMs outperformed all the competing non-linear models (including Random Forest, XGboost, KNN and decision trees) on the same problem of predicting KOA. This finding highlights that the power of the proposed technique lies on the selection of robust and informative risk factors, whereas the complexity of the finally employed classification models plays a less crucial role.

The performance of the proposed FS methodology was compared with eight well-known FS techniques in the recent literature. GenWrapper employing SVM led to the overall best performance (71.25% at 35 selected features), significantly outperforming all the competing algorithms. Specifically, it proved to be more accurate than the classical wrapper FS (which was the second-best approach), and this superiority was more evident for a small number of selected features (up to 20). GenWrapper was also much more effective (at least 3.4% more accurate) than the other seven competing FS techniques (CFS, ILFS, Inf-FS, LASSO, Mrmr, PCA and hybrid). Finally, apart from being the most accurate approach, GenWrapper was prove to also be the most consistent FS technique, with the great majority of the obtained 10FCV accuracies being higher than 70%, whereas all the other competing FS algorithms led to inferior and less consistent accuracies.

During our study, we utilized multimodal data and we managed to identify the variables that mainly contributed to the predictive ability of our models. Important predictive risk factors selected by our models included assessments of pain and function, qualitative assessments of X-rays, assessments of behavioral characteristics, medical history and nutrition from the Center for Epidemiologic Studies Depression Scale (CES-D) and Block Brief 2000 questionnaires. The strongest indicator variables are reporting on knee baseline radiographic OA status (P01SVLKOST), on anthropometric characteristics (P01BMI) and on nutritional (V00SUPCA) and behavioral habits (V00KQOL4). Previous studies [17,19] have also reported similar key predicted variables for KOA progression. Our findings suggest that early functional, behavioral and nutritional interventions should be encouraged and implemented for the prevention or slowing-down of KOA progression.

Genetic algorithms might be costly in computational terms since the evaluation of each individual requires the training of a model. Due to its stochastic nature, the proposed FS takes a longer time to converge, and this could be considered as a limitation. However, the identification of risk factors for KOA progression is, in principle, an offline approach, and therefore, its current execution time (~5 min) is not prohibitive. In the current study, time execution is not considered as crucial as the predictive capability of the finally selected features that can be used to enhance our understanding of whether a patient is at increased risk of progressive KOA. GenWrapper improves the current state of the art by identifying risk factors that are more accurate compared to the ones selected by eight well-known FS algorithms (by at least 3.4%) and, most importantly, more robust in terms of their performance on the entire population of subjects (as it has been validated with an extensive validation mechanism that involved 100 training runs on different data samples). This stated improvement could (i) allow preventive actions to be planned and implemented and (ii) enable more personalized treatment pathways and interventions for treatment, targeting specific risk factors. From a different perspective, being able to identify non-progressors could also prevent over-investigations and over-treatment.

Future work includes the identification of subpopulations of patients that have a greater risk of developing knee OA as well as a higher chance to progress faster. Moreover, quantification of KOA progression is another field that has not been adequately investigated by the scientific community. The combination of more advanced AI tools (e.g., Siamese neural networks) with the FS algorithm proposed in this study could form a reliable basis for quantifying KOA progression.

## 5. Conclusions

This paper focuses on the identification of important and robust risk factors which contribute to KOA progression. The proposed FS methodology relies on an evolutionary machine learning methodology that leads to the selection of a relatively small feature subset (35 risk factors) which generalizes well on the whole dataset (mean accuracy of 71.25%). We investigated the effectiveness of the proposed approach in a comparative analysis with well-known FS techniques with respect to metrics related to both prediction accuracy and generalization capability. The nature of the selected features along with their impact on the prediction outcome (via SHAP) were also discussed to increase our understanding of their effect on KOA progression. Identifying and understanding the contribution of risk factors on KOA progression may enable the implementation of better prevention strategies prioritizing non-surgical treatments, essentially preventing an epidemic of KOA.

## Figures and Tables

**Table 1:** Main categories of the feature subsets considered in this paper. A brief description is given along with the number of features considered per category and for each of the two visits.

Category	Description	Number of Features from Baseline	Number of Features from Visit 1
Subject characteristics	Includes anthropometric parameters (Body mass index (BMI), height, etc.)	36	9
Symptoms	Questionnaire data regarding arthritis symptoms and general arthritis or health-related function and disability	120	80
Behavioral	Includes variables of participants’ quality level of daily routine and social behavior	61	43
Medical history	Questionnaire results regarding a participant’s arthritis-related and general health histories and medications	123	51 (only medications)
Medical imaging outcome	Medical imaging outcomes (e.g., joint space narrowing and osteophytes)	21	-
Nutrition	Block Food Frequency questionnaire	224	-
Physical activity	Questionnaire data regarding leisure activities, etc.	24	24
Physical exam	Participants’ measurements, including knee and hand exams, walking tests and other performance measures	115	26
**Number of features (subtotal):**		724	233
**Total number of features:**		957	

**Table 2:** Hyperparameters of the optimized GenWrapper algorithm. A brief description of each hyperparameter is provided along with the finally selected value.

Parameter	Description	Selected Value
Population size	Number of individual solutions in the population	50
Number of generations	Maximum number of generations before the algorithm halts	100
Mutation rate	Probability rate of being mutated	0.1
Crossover Fraction	The fraction of the population at the next generation, not including elite children, that the crossover function creates.	0.8
Elite Count	Positive integer specifying how many individuals in the current generation are guaranteed to survive into the next generation	5
StallGenLimit	The algorithm stops if the weighted average change in the fitness function value over StallGenLimit generations is less than Function tolerance	50
Tolerance		1 × 10^−3^

**Table 3:** Comparative analysis with respect to the final selection of features: proposed feature ranking versus the feature subset of the best individual solution in the final generation.

FS Criterion	10FCV Accuracy Performed 10 Times				
	Average	Min	Max	Std	No. of Features
Feature subset extracted from the “best” individual solution of the final generation	70.10%	67.59%	72.04%	1.13%	42
Proposed feature ranking	71.25%	69.22%	73.33%	1.57%	35

**Table 4:** Characteristics of the 35 most informative risk factors as selected by the proposed GenWrapper.

Selected Features	Feature Category	Description
P01BMI, P01HEIGHT	Subject characteristics	Anthropometric parameters including height and BMI
KSXRKN1, V00WOMSTFR, KPLKN1, V00WPLKN2, DIRKN16, V00KOOSYML, V00INCOME	Symptoms	Symptoms related to pain, swelling, stiffness and knee difficulty
V00EDCV, V00KQOL4, V00KQOL2, V00CESD9, CEMPLOY	Behavioral	Participants’ quality level of daily routine and social behavior and social status
V00RXCHOND, V00RA, V00CHNFQCV	Medical history	Questionnaire data regarding a participant’s general health histories and medications
P01SVLKOST	Medical imaging outcome	Medical imaging outcomes (e.g., osteophytes)
V00SUPCA, V00FFQ59, V00FFQSZ13, V00FFQ33, V00SUPB2, V00FFQ12, V00SUPFOL, V00FFQ19	Nutrition	Block Food Frequency questionnaire for daily average, how much each time or for past 12 months
PASE2, PASE6, V00PA130CV	Physical activity	Questionnaire results regarding activities during typical week or past 7 days
RKALNMT, V00lfmaxf, V00rfTHPL, V00lfTHPL, STEPST1, V00rkdefcv	Physical exam	Physical measurements of participants, including tests and other performance measures

**Table 5:** Best performance (mean 10FCV) achieved by all competing FS techniques employing SVM along with the number of selected features in which this accuracy was accomplished.

Approach	Best Accuracy (Mean 10FCV)	Number of Features	Statistical Comparison *	Execution Time (sec) **
GenWrapper	71.25	35	-	311.6
Wrapper	69.79	31	*p* < 0.001	10.2
CFS	61.97	69	*p* < 0.001	0.1
ILFS	63.63	82	*p* < 0.001	0.5
Inf-FS	63.32	35	*p* < 0.001	0.1
Lasso	64.41	94	*p* < 0.001	21.2
Mrmr	67.29	36	*p* < 0.001	2.3
Hybrid	67.85	41	*p* < 0.001	15.5
PCA	65.11	29	*p* < 0.001	<0.1

* Statistical comparison with the proposed GenWrapper. ** All the algorithms were executed on an Intel Core i7-7500 processor, 2.70 GHz CPU (16 GB RAM) using MATLAB 2020b.

## Data Availability

Data from the osteoarthritis initiative (OAI) database (available upon request at https://nda.nih.gov/oai/).

## Appendix A

**Table A1:** Selected features that led to the best overall KOA prediction performance in our study. The features have been ranked according to their impact on the classification result as calculated by SHapley Additive exPlanations (SHAP).

Selected Features	Description	Feature Category
P01SVLKOST	Left knee baseline X-ray: evidence of knee osteophytes	Medical imaging outcome
P01BMI	Body mass index	Subject characteristics
V00SUPCA	Block Brief 2000: average daily nutrients from vitamin supplements, calcium (mg)	Nutrition
V00EDCV	Highest grade or year of school completed	Behavioral
V00FFQ59	Block Brief 2000: ice cream/frozen yogurt/ice cream bars, eat how often, past 12 months	Nutrition
V00KQOL2	Quality of life: modified lifestyle to avoid potentially damaging activities to knee(s)	Behavioral
V00CHNFQCV	Chondroitin sulfate frequency of use, past 6 months	Medical history
V00WOMSTFR	Right knee: WOMAC Stiffness Score	Symptoms
V00FFQSZ13	Block Brief 2000: french fries/fried potatoes/hash browns, how much each time	Nutrition
V00KQOL4	Quality of life: in general, how much difficulty have with knee(s)	Behavioral
P01HEIGHT	Average height (mm)	Subject characteristics
V00lfTHPL	Left Flexion MAX Force High Production Limit	Physical exam
V00rkdefcv	Right knee exam: alignment varus or valgus	Physical exam
V00FFQ19	Block Brief 2000: green beans/green peas, eat how often, past 12 months	Nutrition
V00FFQ33	Block Brief 2000: beef steaks/roasts/pot roast (including in frozen dinners/sandwiches), eat how often, past 12 months	Nutrition
KPLKN1	Left knee pain: twisting/pivoting on knee, last 7 days	Symptoms
PASE2	Leisure activities: walking, past 7 days	Physical activity
V00INCOME	Yearly income	Behavioral
V00PA130CV	How often climb up total of 10 or more flights of stairs during typical week, past 30 days	Physical activity
V00CESD9	How often thought my life had been a failure, past week	Behavioral
PASE6	Leisure activities: muscle strength/endurance, past 7 days	Physical activity
DIRKN16	Right knee difficulty: heavy chores, last 7 days	Symptoms
V00SUPB2	Block Brief 2000: average daily nutrients from vitamin supplements, B2 (mg)	Nutrition
STEPST1	20-meter walk: trial 1 number of steps	Physical exam
V00FFQ12	Block Brief 2000: any other fruit (e.g., grapes/melon/strawberries/peaches), eat how often, past 12 months	Nutrition
KSXRKN1	Right knee symptoms: swelling, last 7 days	Symptoms
V00lfmaxf	Left Flexion MAX Force	Physical exam
V00rfTHPL	Right Flexion MAX Force High Production Limit	Physical exam
RKALNMT	Right knee exam: alignment, degrees (valgus negative)	Physical exam
CEMPLOY	Current employment	Behavioral
V00KOOSYML	Left knee: KOOS Symptoms Score	Symptoms
V00WPLKN2	Left knee pain: stairs, last 7 days	Symptoms
V00RA	Charlson Comorbidity: have rheumatoid arthritis	Medical history
V00SUPFOL	Block Brief 2000: average daily nutrients from vitamin supplements, folate (mcg)	Nutrition
V00RXCHOND	Rx Chondroitin sulfate use indicator	Medical history
